# Supplementary material for: Automated Skull Stripping in Mouse Functional Magnetic Resonance Imaging Analysis Using 3D U-Net
Source: Front Neurosci. 2022 Mar 10;16:801769. doi: 10.3389/fnins.2022.801769 (PMC8965644; doi:10.3389/fnins.2022.801769)

Table S1 – A total of 27 components extracted by the group ICA analysis and corresponding brain regions.


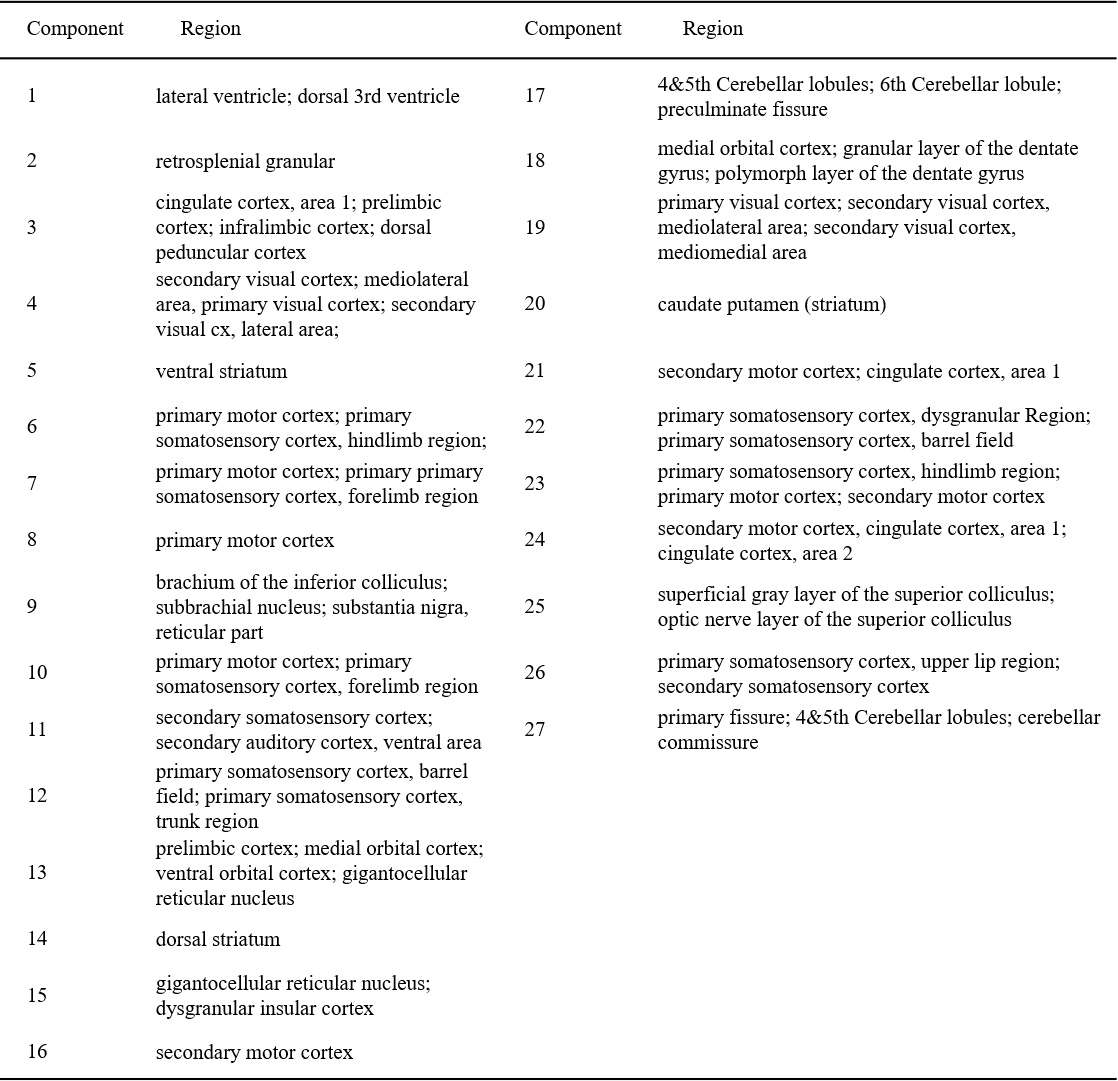

Supplement: Supplementary file 1 [file Table_1.DOCX]
