# Supplementary figures and images for: Automated Skull Stripping in Mouse Functional Magnetic Resonance Imaging Analysis Using 3D U-Net
Source: Front Neurosci. 2022 Mar 10;16:801769. doi: 10.3389/fnins.2022.801769 (PMC8965644; doi:10.3389/fnins.2022.801769)

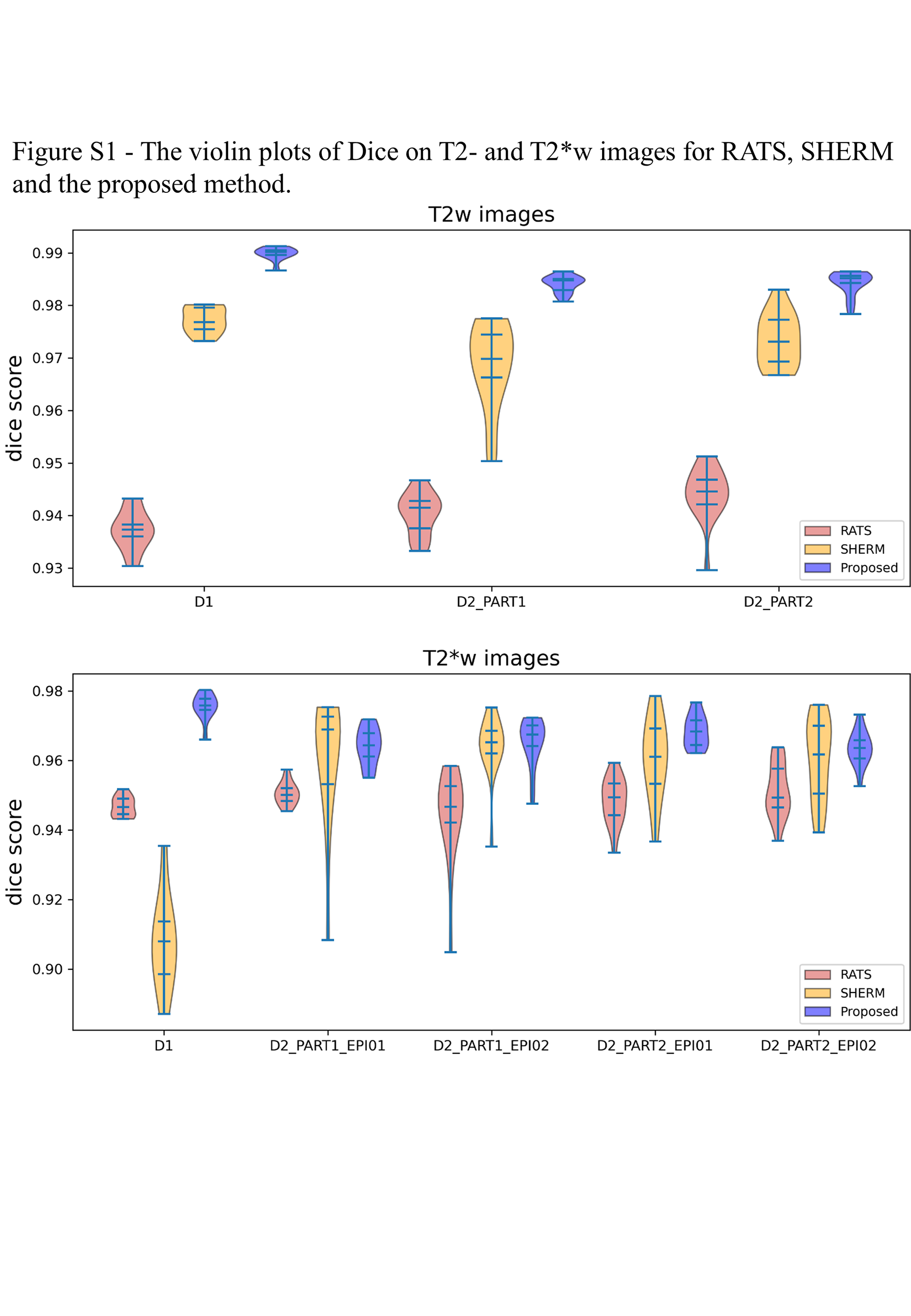

Supplement: Supplementary file 2 [file Image_1.TIF]
